# Use of Nursing Support Among Nurses for Caregiver Burden in Family Caregivers of Terminally Ill Patients with Cancer in Palliative Care Units in Japan: Multisite Cross-Sectional Study

**DOI:** 10.1089/pmr.2024.0043

**Published:** 2024-10-03

**Authors:** Kohei Kajiwara, Masamitsu Kobayashi, Kimiko Nakano, Yusuke Kanno, Miharu Morikawa, Yoshinobu Matsuda, Jun Kako

**Affiliations:** ^1^ Faculty of Nursing, Japanese Red Cross Kyushu International College of Nursing, Munakata, Japan.; ^2^ Graduate of Nursing Science, St. Luke’s International University, Tokyo, Japan.; ^3^ Nursing department, Mie University Hospital, Tsu, Japan.; ^4^ Nursing Science, Tokyo Medical and Dental University, Tokyo, Japan.; ^5^ Graduate School of Medicine, Kyoto University, Kyoto, Japan.; ^6^ Department of Psychosomatic Internal Medicine, NHO Kinki Chuo Chest Medical Center, Sakai, Japan.; ^7^ Graduate School of Medicine, Mie University, Tsu, Japan.

**Keywords:** burden, cancer, caregiver, nursing support, palliative care

## Abstract

**Purpose:**

This study explores the use of nursing support among nurses for caregiver burden in family caregivers of terminally ill patients with cancer in palliative care units (PCUs).

**Methods:**

Requests were sent to 389 institutions, and cooperation was received from 162 PCUs. Nurses at 162 PCUs were asked to participate in an Internet survey regarding nursing practices for caregiver burden in Japan. The frequency of six nursing support practices (extracted in a scoping review) was reported using a 5-point Likert scale.

**Results:**

The response rate was 22.3% (539/2448). Support for reducing caregiver stress was the most frequently provided nursing support (mean Likert score: 2.41 for monthly prognosis and 2.42 for weekly prognosis). Psychological and educational support was mainly provided via non-face-to-face (telephone) (mean Likert score: 2.26 for monthly prognosis and 2.21 for weekly prognosis) and face-to-face methods (mean Likert score: 2.32 for monthly prognosis and 2.29 for weekly prognosis).

**Conclusion:**

Nursing support was provided through telephone support and face-to-face interactions and aimed at reducing caregiver stress among nurses and family caregivers of patients with terminal cancer in PCUs. In this study, the trends in nursing support were similar for patients with a prognosis of weeks or months.

## Introduction

Family members of patients with terminal cancer experience stress associated with the deterioration of the patient’s condition,^1–3^ leading to the need for caregiver support. Due to the nature of terminal cancer, treatment of the underlying disease or pathology can be challenging, with palliative care often serving as the mainstay of support. Previous studies have reported an increased burden on family caregivers due to the progression of symptoms in patients with cancer^4^ and the emergence of symptoms in the terminal stage, contributing to caregiver burden.^5^ Therefore, support focusing on caregiver burden is important.

In palliative care, nursing support, also known as nursing interventions, is a prominent nonpharmacological intervention. Despite extensive research regarding family caregivers of patients with cancer has been conducted, no guidelines exist. Palliative care literature reviews have highlighted the importance of family support.^6^ Moreover, previous studies have highlighted the importance of caregiver burden in palliative care and palliative care units (PCUs).^7–9^ However, research regarding nursing support for caregiver burden to family caregivers of terminally ill patients with cancer in PCUs is insufficient.

In clinical practice, the best available research evidence is integrated with family preferences, the practice knowledge of care environments, and care providers to deliver optimal health care. The provision of nursing support to family caregivers of terminally ill patients with cancer is primarily guided by the demands of family caregivers and the practice knowledge of care providers due to the limited availability of relevant research. A previous study explored the impact of nursing support on caregiving burden by conducting a comprehensive scoping review.^10^ However, the specific support provided by nurses and the extent of the support remain unclear. While extensive evidence has been presented, revealing the discrepancy between the evidence and its use of nursing support is crucial for effectively translating the evidence into practice.

## Study Purpose

This study explores the use of nursing support among nurses for caregiver burden in family caregivers of terminally ill patients with cancer in PCUs.

## Materials and Methods

### Participants and study design

Specialized palliative care in Japan includes palliative care wards and teams,^11^ and this survey specifically targeted nurses working in specialized palliative care. In recruiting the participants, out of all 389 facilities in Japan that have filed notifications for PCUs (as of September 14, 2023), this study requested participation from 389 facilities and obtained cooperation from 162 facilities. This study involved nurses from 162 PCUs in Japan. Research explanation documents and participation request letters were mailed to the directors of nursing in PCUs, requesting the cooperation of their nurses. During October 2023–March 2024, the nurses completed an anonymous Internet survey.

### Measurements

The Internet survey was conducted based on the results of a scoping review. The scoping review, conducted prior to this study, aimed to extract evidence regarding nursing support provided to family caregivers of terminally ill patients with cancer.^10^ Seven components of nursing support and caregiver burden for family members were identified.^10^ In addition, using the results of the Delphi method^12^ as a reference, we conducted a preliminary survey with nine experienced PCU nurses to extract the different components of nursing support. The result of the survey identified nursing support components: psychological and educational support using mainly non-face-to-face methods (ICT; Information and Communication Technology), psychological and educational support mainly using non-face-to-face methods (telephone), face-to-face psychological and educational support, support for reducing caregiver stress, mindfulness to support both patients and caregivers, and support for both patients and caregivers. The use of these support methods for family caregivers of patients with cancer, with a prognosis of weeks or months, was assessed using a 5-point Likert scale (1: rarely, 2: seldom, 3: sometimes, 4: frequently/often, and 5: very frequently/very often). Demographic data, such as sex, age, years of nursing experience, years of PCU experience, educational background, and qualifications, were collected from the survey respondents.

### Statistical analyses

The outcome was the frequency of the use of nursing support for caregivers of patients with a monthly and weekly prognosis. Demographic information and nursing support data are summarized using descriptive statistics. Mean scores and standard deviations were calculated for Likert scale responses to summarize both demographic information and nursing support data. All statistical analyses were performed using EZR, a component of R software (Saitama Medical Center, Jichi Medical University, Saitama, Japan) that includes statistical functions frequently used in biostatistics.^13^

### Research ethics

This research was approved by the Clinical Research Ethics Review Committee of Mie University Hospital (U2023-011). All participants provided consent to participate in the study.

## Results

The survey response rate was 22.3% (539/2448). The respondents were predominately female (*n* = 511; 94.8%) with a mean age of 42.4 years (standard deviation [SD]: 9.8 years) (Table 1). Most respondents had attended nursing vocational school (including junior college) (*n* = 456; 84.6%). The mean number of years of nursing experience was 18.6 years (SD: 9.6 years), and the mean number of years of experience in the PCU was 4.8 years (SD: 4.3 years). The majority of participants with specialty certifications were palliative care nurses (*n* = 29; 5.3%).

**Table 1.** Participant Characteristics (*n* = 539)

	*n* (%)
Sex	
Female	511 (94.8)
Male	26 (4.8)
Prefer not to answer	2 (0.4)
Educational Background	
Nursing vocational school (including junior college)	456 (84.6)
Nursing university	64 (11.9)
Master’s program (Predoctoral program)	10 (1.9)
Doctoral program (Postdoctoral program)	1 (0.2)
Qualifications	
Certified Nurse in Palliative Care	29 (5.3)
Certified Nurse in Cancer Pain Management Nursing	4 (0.7)
Certified Nurse Specialist in Cancer Nursing	3 (0.6)
Qualifications (Other)	
End-of-Life Care Specialist	8 (1.5)
Certified Public Psychologist	2 (0.4)
Other	6 (1.1)

	**Mean (SD)**
Age (years)	42.4 (9.8)
Years of nursing experience	18.6 (9.6)
Years of experience in the palliative care unit	4.8 (4.3)

SD, standard deviation.

Support for reducing caregiver stress was the most frequently provided nursing support (mean Likert score: 2.41 for monthly prognosis and 2.42 for weekly prognosis) (Table 2). Psychological and educational support was mainly provided via non-face-to-face (telephone) (mean Likert score: 2.26 for monthly prognosis and 2.21 for weekly prognosis) and face-to-face methods (mean Likert score: 2.32 for monthly prognosis and 2.29 for weekly prognosis). Psychological and educational support was provided using mainly non-face-to-face methods (ICT) (mean Likert score: 1.96 for monthly prognosis and 1.93 for weekly prognosis), and support for both patients and caregivers (mean Likert score: 2.04 for monthly prognosis and 2.05 for weekly prognosis) was occasionally provided to family caregivers. Mindfulness was not frequently used to support both patients and caregivers (mean Likert score: 1.47 for monthly prognosis and 1.46 for weekly prognosis).

**Table 2.** Nursing Support to Family Caregiver of Patients with Terminal Cancer (*n* = 539)

	*n* (%)	
	Prognosis of months	Prognosis of weeks
Psychological and educational support using mainly non-face-to-face (ICT)		
Rarely	275 (51.2)	279 (52.0)
Seldom	94 (17.5)	97 (18.1)
Sometimes	100 (18.6)	97 (18.1)
Frequently/Often	48 (8.9)	48 (8.9)
Very frequently/Very often	20 (3.7)	16 (3.0)
No response	2 (0.4)	2 (0.4)
Psychological and educational support mainly using non-face-to-face (telephone) methods		
Rarely	217 (40.4)	220 (41.0)
Seldom	76 (14.2)	97 (18.1)
Sometimes	151 (28.1)	131 (24.4)
Frequently/Often	71 (13.2)	65 (12.1)
Very frequently/Very often	22 (4.1)	24 (4.5)
No response	2 (0.4)	2 (0.4)
Mainly face-to-face psychological and educational support		
Rarely	184 (34.3)	185 (34.5)
Seldom	104 (19.4)	114 (21.2)
Sometimes	164 (30.5)	157 (29.2)
Frequently/Often	63 (11.7)	60 (11.2)
Very frequently/Very often	22 (4.1)	21 (3.9)
No response	2 (0.4)	2 (0.4)
Support for reducing caregiver stress		
Rarely	175 (32.6)	176 (32.8)
Seldom	104 (19.4)	102 (19.0)
Sometimes	151 (28.1)	143 (26.6)
Frequently/Often	78 (14.5)	87 (16.2)
Very frequently/Very often	29 (5.4)	29 (5.4)
No response	2 (0.4)	2 (0.4)
Mindfulness to support both patients and caregivers		
Rarely	399 (74.3)	398 (74.1)
Seldom	62 (11.5)	70 (13.0)
Sometimes	47 (8.8)	40 (7.4)
Frequently/Often	21 (3.9)	20 (3.7)
Very frequently/Very often	8 (1.5)	9 (1.7)
No response	2 (0.4)	2 (0.4)
Support for both patients and caregivers		
Rarely	241 (45.0)	242 (45.1)
Seldom	111 (20.7)	116 (21.6)
Sometimes	121 (22.6)	108 (20.1)
Frequently/Often	47 (8.8)	53 (9.9)
Very frequently/Very often	16 (3.0)	18 (3.4)
No response	3 (0.6)	2 (0.4)

## Discussion

This study investigated the use of nursing support among nurses for caregiver burden in family caregivers of patients with terminal cancer in PCUs. This study provided nursing telephone support and face-to-face interactions, with the aim of reducing caregiver stress. Family caregivers did not often receive support through mindfulness. This study’s results differ from those of a previous scoping review regarding caregiver burden.^10^

In this study, support provided to family caregivers included telephone support, face-to-face interactions, and interventions aimed at reducing caregiver stress. A previous study found high applicability results for telephone support and interventions aimed at reducing caregiver stress.^12^ Non-face-to-face methods and mindfulness were used less frequently, which is consistent with the results of a previous study.^12^ These results may be due to the need for appropriate equipment and preparation for such support methods, reflecting the convenience highlighted in previous studies.^14^ Meaningful support using non-face-to-face methods is dependent on the supporter’s experience.^15^ The importance of mindfulness training has been reported in a previous study.^16^ Support through mindfulness is beneficial; however, this knowledge may not be adequately disseminated at the practical level. Considering caregiver burden on caregivers and advancements in technology, we believe that ICT and telephone support can be further developed to support caregiver burden. Therefore, support mechanisms that are currently being underutilized must be investigated in the future to facilitate the use of nursing support. Additionally, we believe that it is imperative to consider research on the implementation of evidence-based nursing support.

In this study, the trends in nursing support were similar for patients with a prognosis of weeks or months. This is consistent with a previous study that reported no significant difference in the treatment and terminal phases of illnesses.^10^ Caregivers often demand continuous support to acquire the necessary knowledge and skills for each stage of cancer care,^17^ which may be because caregiver burdens do not directly affect the terminal phase.

## Limitations

This study is not without limitations. First, the response rate was low and the respondents could have been more enthusiastic about using nursing support. Therefore, the generalizability of our results is limited. Studies using postal methods with individual nurses may be effective in improving response rates. Second, the use of nursing support may be generalized in other countries. Third, since this study was a self-administered survey, there could be self-reporting bias. Our findings may differ from the actual nursing support provided. Future research investigating the actual nursing support provided for caregiver burden is warranted.

## Conclusions

The primary method of nursing support was telephone support and face-to-face interactions and aimed at reducing caregiver stress for family caregivers of patients with terminal cancer. The trends in nursing support were similar for patients with a prognosis of weeks or months. Further research is necessary to develop and implement nursing support strategies. Moreover, subsequent research should prioritize the development and evaluation of structured nursing support programs that integrate both ICT and telephone interactions to alleviate caregiver stress.

## Data Availability

The data are registered in the individual case data repository of the UMIN (University Hospital Medical Information Network) in Japan.

## References

[1] Breitbart W, Gibson C, Tremblay A. The delirium experience: Delirium recall and delirium-related distress in hospitalized patients with cancer, their spouses/caregivers, and their nurses. Psychosomatics 2002;43(3):183–194; doi: 10.1176/appi.psy.43.3.183 12075033

[2] Osse BH, Vernooij-Dassen MJ, Schadé E, et al. Problems experienced by the informal caregivers of cancer patients and their needs for support. Cancer Nurs 2006;29(5):378–388; doi: 10.1097/00002820-200609000-00005 17006111

[3] Kent EE, Rowland JH, Northouse L, et al. Caring for caregivers and patients: Research and clinical priorities for informal cancer caregiving. Cancer 2016;122(13):1987–1995; doi: 10.1002/cncr.29939 26991807 PMC5597246

[4] Genderson MW, Thomson MD, Siminoff LA. Where you begin is not necessarily where you end: The mental and physical health trajectories of cancer caregivers over time. Support Care Cancer 2024;32(4):233; doi: 10.21203/rs.3.rs-3513142/v1 38499880

[5] Jung MH, Park MH, Kim SJ, et al. Delirium-related knowledge, caregiving performance, stress levels, and mental health of family caregivers of terminal cancer patients with delirium in a hospice care unit. J Hosp Palliat Care 2021;24(2):116–129; doi: 10.14475/jhpc.2021.24.2.116 37675238 PMC10180047

[6] Alam S, Hannon B, Zimmermann C. Palliative care for family caregivers. J Clin Oncol 2020;38(9):926–936; doi: 10.1200/JCO.19.00018 32023152

[7] Axelsson L, Alvariza A, Holm M, Årestedt K. Intensity of predeath grief and postdeath grief of family caregivers in palliative care in relation to preparedness for caregiving, caregiver burden, and social support. Palliat Med Rep 2020;1(1):191–200. 34223476 10.1089/pmr.2020.0033 PMC8241336

[8] Ahmad Zubaidi ZS, Ariffin F, Oun CTC, Katiman D. Caregiver burden among informal caregivers in the largest specialized palliative care unit in Malaysia: A cross sectional study. BMC Palliat Care 2020;19(1):186. 33292214 10.1186/s12904-020-00691-1 PMC7722979

[9] Tanco K, Prado B, Qian Y, et al. A comparison of caregiver burden of patients with advanced cancer in different palliative cancer care settings. J Palliat Med 2021;24(12):1766–1775. 33926226 10.1089/jpm.2021.0026

[10] Kajiwara K, Kobayashi M, Morikawa M, et al. Nursing support for caregiver burden in family caregivers of patients with cancer: A scoping review. Am J Hosp Palliat Care 2023:10499091231215808; doi: 10.1177/10499091231215808 PMC11367804 37963324

[11] Morita T, Kizawa Y. Palliative care in Japan: A review focusing on care delivery system. Curr Opin Support Palliat Care 2013;7(2):207–215; doi: 10.1097/SPC.0b013e3283612241 23635880

[12] Kako J, Kajiwara K, Kobayashi M, et al. Applicability of nursing support for patients with terminal cancer and their families: A Delphi study. Am J Hosp Palliat Care 2024:10499091241245266; doi: 10.1177/10499091241245266 38580325

[13] Kanda Y. Investigation of the freely-available easy-to-use software “EZR” (Easy R) for medical statistics. Bone Marrow Transplant 2013;48(3):452–458; doi: 10.1038/bmt.2012.244 23208313 PMC3590441

[14] Laidsaar-Powell R, Giunta S, Butow P, et al. Development of web-based education modules to improve carer engagement in cancer care: Design and user experience evaluation of the e-Triadic Oncology (eTRIO) modules for clinicians, patients, and carers. JMIR Med Educ 2024;10:e50118; doi: 10.2196/50118 38630531 PMC11063882

[15] Albright L, Ko W, Buvanesh M, et al. Opportunities and challenges for augmented reality in family caregiving: Qualitative video elicitation study. JMIR Form Res 2024;8:e56916; doi: 10.2196/56916 38814705 PMC11176885

[16] Schellekens MPJ, van den Hurk DGM, Prins JB, et al. Mindfulness-based stress reduction added to care as usual for lung cancer patients and/or their partners: Aa multicentre randomized controlled trial. Psychooncology 2017;26(12):2118–2126; doi: 10.1002/pon.4430 28337821

[17] Bahrami M, Sebzari AR, Nasiri A. Caregivers’ demands: Caring atmosphere expected by cancer patients’ caregivers-a qualitative content analysis. Support Care Cancer 2024;32(6):389; doi: 10.1007/s00520-024-08575-3 38802620

